# Sesn2/AMPK/mTOR signaling mediates balance between survival and apoptosis in sensory hair cells under stress

**DOI:** 10.1038/cddis.2017.457

**Published:** 2017-10-05

**Authors:** Daniel Bodmer, Soledad Levano-Huaman

**Affiliations:** 1 Department of Biomedicine, University of Basel Hospital, Basel, Switzerland; 2 Department of Otolaryngology, Head and Neck Surgery, University of Basel Hospital, Basel, Switzerland

Sensory hair cells located in the inner ear are essential for detecting sounds. Hair cells can be damaged by noise exposure, aminoglycoside antibiotics, chemotherapeutic agents and effects of aging. In mammals, hair cells do not regenerate. The greater the loss of hair cells, the more severe is the hearing loss. Use of hearing aids can ameliorate hearing sensitivity. However, when large portion of hair cells are damaged or missing, the use of hearing aid may be of limited help. Our efforts in designing strategies to preserve sensory hair cells depend on understanding the mechanism underlying hair cell survival and death. Hair cells can activate different types of survival or apoptotic factors that are determined by the strength and nature of stressors. Gentamicin, an aminoglycoside antibiotic, can induce damage to hair cells in the inner ear. Gentamicin is employed in clinical practice to treat severe or serious bacterial infections especially in third world countries. Serious side effects of gentamicin can include hearing loss and nephrotoxicity. Increasing evidence suggest that hair cell death induced by gentamicin occur through apoptosis. In the last years, significant progress has been made in understanding the process of hair cell damage and loss induced by aminoglycoside. Formation of reactive oxygen species (ROS) has been observed in inner ear explants exposed to gentamicin.^1^ ROS are produced in normal cellular metabolism, however excessive generation of ROS by gentamicin leads to oxidative stress in sensory hair cells and neurons. Reduction of inner ear damage by gentamicin has been achieved using free radical scavengers and antioxidants.^2^ Sestrins are highly conserved stress-responsive proteins and are known to protect cells against oxidative stress and aging as well as regulate cell growth and viability. Protective role of sestrin-2 (Sesn2), member of the oxidative stress pathway, has been postulated in many conditions, including age-related pathologies especially in the heart, liver and nervous system.^3, 4, 5, 6^ However, until now, the role of sestrins in the inner ear has never been studied. The recent characterization of the protein structure of human Sesn2 increases the understanding of their several antioxidant mechanisms.^7^ One of the mechanisms of Sesn2 to prevent oxidative stress is the activation of the nuclear factor erythroid 2-related factor 2 (Nrf2) pathway leading to induction of antioxidant proteins.^8^ Data from Nrf2-KO mice demonstrated that Nrf2 protects hair cells against gentamicin and age-related hearing loss by upregulating antioxidant enzymes.^9^ Another antioxidant mechanism of Sesn2 involves the regulation of mammalian target of rapamycin complex 1 (mTORC1). Sesn2 can negatively regulate mTORC1 via AMP-activated protein kinase (AMPK) and recombinant activating genes (Rag), and thus attenuates ROS accumulation.^8^ Several studies have shown that the regulation of AMPK activation by Sesn2 influences cell survival and function in different organs. For example, Sesn2-deficient mice have shown an impaired activation of cardiac AMPK and their hearts presented larger myocardial infarcts compared to their wild-type littermates.^6^ Furthermore, AMPK reactivation in Sesn2-KO mice restored hepatic insulin sensitivity.^5^ AMPK is likewise activated in the cochlear spiral ligament following acoustic overstimulation.^10^ Finally, depletion of Sens2 by RNA interference resulted in reduction of autophagy.^11^ Whether the stress caused by gentamicin in auditory hair cells also implicate Sesn2 is unknown. Sesn2 may activate AMPK, may inhibit mTOR, may lead autophagy induction and therefore may stimulate the survival of hair cells after aminoglycoside exposure. To investigate this, we conducted a study published recently in *Cell Death Discovery*.^12^ We found that Sesn2 is involved in the protection of hair cells against gentamicin and the loss of Sesn2 increased the susceptibility of hair cells to gentamicin (Figure 1). We localized the expression of Sens2 in auditory hair cells and spiral ganglion neurons. At early time points after gentamicin exposure, Sesn2 expression was stable in treated organ explants of wild-type mice. After 24 h of gentamicin exposure, Sesn2 expression in explants of wild-type mice was significantly reduced. At this time, explants of both genotypes showed an increased hair cell death rate in gentamicin-exposed explants compared to those control explants. Interestingly, gentamicin-exposed explants from Sesn2-KO mice displayed greater hair cell loss compared to those from wild-type explants. In addition, higher levels of stained apoptotic cells were found in gentamicin-exposed explants from Sesn2-KO mice than in wild-type mice. Protein expression analysis of Sesn2 stress-responsive pathways displayed a decrease in AMPK activation and increase in mTORC1 activation in explants from mice of both genotypes after gentamicin exposure. In addition, explants of Sesn2-KO mice displayed low levels of AMPK phosphorylation and high levels of mTOR activation; this basal dysregulation of AMPK/mTOR axis seems to favor the mechanism of apoptosis after gentamicin exposure. Similar to our observations in the inner ear, the crosstalk between AMPK and mTOR was disrupted in Sesn2-deficient hepatic cells and liver tissue. These deficient cells were highly sensitive to endoplasmatic reticulum (ER) stress-induced cell death, however, the pathologies linked to ER stress were suppressed by reconstitution of Sens2, adding an AMPK activator or mTOR inhibitor.^13^ In our recent study, we also showed a significant increase in hair cell survival after gentamicin exposure by using rapamycin, a known inhibitor of mTORC1 activation.^12^ Rapamycin, not only attenuated gentamicin-induced hair cell damage as demonstrated in our study, but also attenuated noise-induced hair cell loss. Yuan *et al.*^14^ found that treatment with rapamycin increased the expression of autophagy marker microtubule-associated protein light chain 3B by blocking mTORC1, reducing the oxidative stress marker 3-nitrotyrosine, and thus preventing noise-induced hearing loss and hair cell death.

It is worth noting that Sesn1 and Sesn3 seem not to compensate the loss of Sesn2 expression.^12^ Upregulation of Sesn2 expression was apparently not essential for auditory hair cell protection against gentamicin, in contrast to the early observations of upregulation of Sesn2 mRNA and protein in peripheral nerves after injury.^3^ The availability of Sesn2 may be required during gentamicin-induced stress; accordingly, auditory hair cells of Sesn2-KO mice were more susceptible to gentamicin exposure than those of the wild-type mice.

Collectively, Sesn2 contribute to the regulation of AMPK/mTOR signaling and protects hair cells against the induction of cell death by gentamicin. Dysregulation of AMPK/mTOR leads to impaired autophagy. The identification of the Sens2/AMPK/mTOR signaling pathway in hair cells as one of the survival strategies against gentamicin-induced stress will disclose new potential therapeutic targets for ototoxicity.

## Figures and Tables

**Figure 1:**
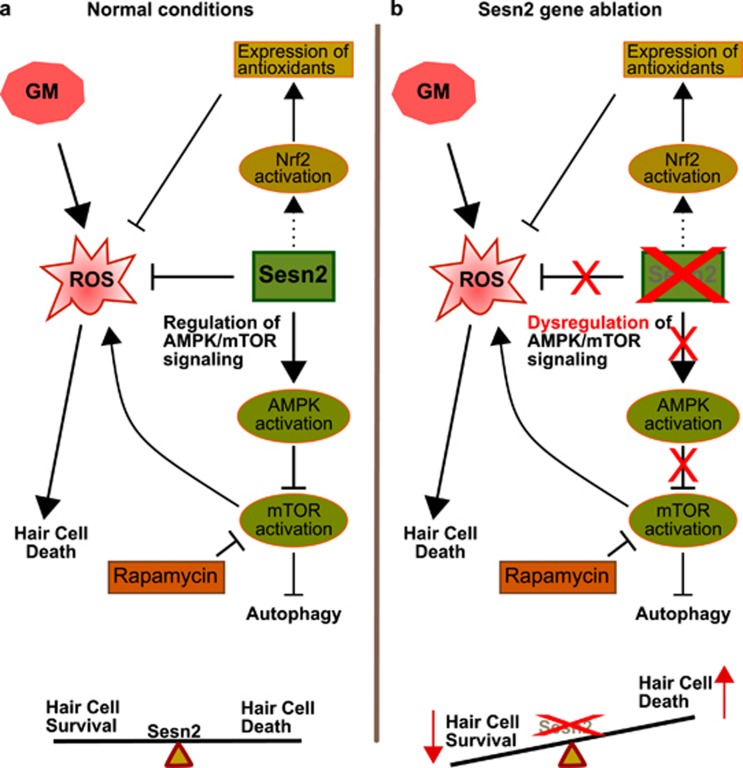
Model illustrating the potential mechanism by which Sesn2 regulates survival of sensory hair cells following gentamicin exposure. (**a**) Sesn2 prevents oxidative stress and reduces ROS levels through activation of Nrf2 and inhibition of mTOR activation.^9, 12^ We found that Sesn2 regulates the crosstalk between AMPK and mTOR in hair cells.^12^ Sesn2 is an important player in the regulation of cell fate after gentamicin exposure. (**b**) Dysregulation of AMPK/mTOR signaling in absence of Sesn2.^12^ Hair cells lacking Sesn2 are more sensitive to gentamicin. We found high rate of hair cell death after gentamicin exposure. Rapamycin attenuates gentamicin-induced hair cell damage. The activation of Nrf2 by Sesn2 will need to be confirmed in sensory hair cells
